# Age adjustment of cancer survival rates: methods, point estimates and standard errors

**DOI:** 10.1038/sj.bjc.6602704

**Published:** 2005-07-19

**Authors:** H Brenner, T Hakulinen

**Affiliations:** 1 Department of Epidemiology, German Centre for Research on Ageing, Bergheimer Strasse 20, D-69115 Heidelberg, Germany; 2 Finnish Cancer Registry, Institute for Statistical and Epidemiological Cancer Research, Liisankatu 21 B, FIN-00170 Helsinki, Finland

**Keywords:** age adjustment, cancer registries, survival

## Abstract

We empirically evaluated the performance of a new method for age adjustment of cancer survival compared to traditional age adjustment using data from the Finnish Cancer Registry. We find that both methods provide almost identical results for absolute survival but the new method generally provides more meaningful estimates of relative survival with often a smaller standard error.

Five or 10-year are commonly reported outcome measures by cancer registries. Because prognosis strongly depends on age for many forms of cancer, age adjustment is often employed in comparative analyses such as international comparative survival studies. However, traditional age adjustment, which is based on calculating a weighted average of age-specific survival with weights reflecting the age distribution of cancer patients in some (typically site-specific) standard cancer population (Verdecchia *et al*, 1999), is often hindered by sparseness of data within certain age groups. Furthermore, it has been shown that traditional age adjustment may substantially shift relative survival rates downward even if the standard population has the same age distribution as the study population (Brenner and Hakulinen, 2003).

Recently, an alternative method of age adjustment has been proposed to overcome both the practical difficulties and the conceptual inconsistencies in age adjustment of survival (Brenner *et al*, 2004). In this paper, we provide a comparative empirical assessment of point estimates of absolute and relative survival rates and their standard errors obtained with the traditional and the alternative methods.

## MATERIALS AND METHODS

Our analysis is based on data from the Finnish Cancer Registry, which covers the whole population of Finland (about 5.3 million people) and is well recognised for its high data quality and completeness (Teppo *et al*, 1994). Our empirical analysis was carried out among patients aged 15 years or older who were diagnosed with one of 15 common forms of cancer in 1989 and followed with respect to vital status until the end of 1999. The site-specific EUROCARE-2 cancer populations, reflecting the age distribution of patients diagnosed in 1985–1989 and included in the comparative analyses of cancer survival in Europe within the EUROCARE-2 project (Berrino *et al*, 1999), were used as standard populations for age adjustment.

We first carried out descriptive analyses of the age distribution of the Finnish cancer patients and the EUROCARE-2 standard cancer populations. We then calculated age-specific absolute and relative survival rates for each cancer site. Relative survival rates were calculated as the absolute survival rates divided by the expected survival rates in the absence of cancer (Ederer *et al*, 1961). The latter were derived from age-, sex- and calendar year-specific life tables of the general Finnish population using Hakulinen's (1982) method.

Next, we calculated crude and age-adjusted absolute and relative survival rates and their standard errors. Age adjustment to the EUROCARE-2 standard cancer populations was performed both by the traditional method and by the alternative method recently proposed by Brenner *et al* (2004). With the former method, a weighted average of age-specific survival rates is calculated, whereas with the latter method, age-specific weights are individually assigned to patients in the first place, and one then carries out conventional survival analysis using the weighted individual data.

As in the EUROCARE project, the following five age groups were used for age adjustment with both methods: 15–44, 45–54, 55–64, 65–74 and 75+ years. For prostate cancer, which is extremely rare among younger and middle-aged adults, the age groups were 15–54, 55–64, 65–74, 75–84, and 85+ years. The usual formula (based on large sample approximations) for the standard error of traditionally adjusted survival rates often cannot be applied due to lack of data within single age strata (Verdecchia *et al*, 1999). Also available formulae may often substantially overestimate standard errors of relative survival rates (Brenner and Hakulinen, 2005). Formulae for straightforward estimates of the standard error of the alternatively adjusted survival rates are yet to be developed for these reasons, standard errors of all types of survival rates were determined empirically by bootstrap analysis as the standard deviation of the respective point estimates in 10 000 bootstrap samples for each cancer site (Efron and Tibshirani, 1986).

All analyses were performed with publicly available SAS macros for both absolute and relative survival analysis (Arndt *et al*, 2004; Brenner *et al*, 2004), which were extended to allow for bootstrap analyses.

## RESULTS

Table 1 shows the numbers and age distribution of patients included in this analysis by cancer site. The most frequent cancer in Finland in 1989 was breast cancer, followed by lung, prostate, stomach and colon cancers. The age distribution strongly varied by cancer site. Relatively high proportions of patients with thyroid and cervical cancer and melanoma were below age 45 years, whereas almost half of the patients with prostate cancer were 75 years or older at the time of diagnosis. For most cancers, the age distribution of Finnish patients was not too different from the age distribution of the EUROCARE standard population. For some cancers (cancers of the oral cavity, cervix and corpus uteri), however, the proportion of older patients was much higher in Finland, which underlines the importance of age adjustment if survival comparisons are made even within Europe.

As Table 2 shows, a strong age gradient with lower rates among older than among younger patients was seen for both absolute and relative survival for most forms of cancer. The age gradient was particularly large for patients with cancers of the uterine cervix, the ovaries, the urinary bladder and the thyroid gland. No clear age gradient was seen for relative survival of patients with stomach and breast cancers, and relative survival was higher in the oldest than in the youngest age group in prostate cancer.

The crude and age-adjusted absolute survival rates and their standard errors are given in Table 3. Both the point estimates and their standard errors were virtually identical for both methods of age adjustment. Adjusted point estimates were quite similar to the crude ones for those cancer sites with a similar age distribution in Finland and the EUROCARE population. Age-adjusted 5- and 10-year absolute survival rates were considerably higher than the crude ones for those cancers with a higher proportion of younger patients in the standard population and a strong age gradient in prognosis (cancers of the oral cavity, cervix and corpus uteri).

The crude and age-adjusted relative survival rates and their standard errors are given in Table 4. Unlike the age-adjusted absolute survival rates, they often differed according to the method of age adjustment. The differences were mostly modest for 5-year relative survival estimates, but substantial differences were seen for some of the 10-year relative survival estimates (point estimates up to 6.7% units lower, standard errors up to 1.1% units or, in relative terms, 40% higher with the traditional method compared to the alternative method). With traditional age adjustment, the adjusted 10-year relative survival estimates were quite close to or lower than the crude estimates, even when the age distribution was shifted towards the younger ages as, for example, in oral cavity and bladder cancers. In other cases (e.g. thyroid cancer), traditional age adjustment substantially reduced 10-year relative survival estimates, despite very similar age distributions of cancer patients in the standard population and in the study population. No such patterns were seen with alternative age adjustment. In addition, standard errors of age-adjusted relative survival were often substantially lower with the alternative age adjustment than with the traditional age adjustment, particularly for 10-year relative survival.

## DISCUSSION

In our previous work, the alternative method for age adjustment had been empirically illustrated for point estimates of relative survival for one single cancer site only (Brenner *et al*, 2004). The present analysis extends these findings to both point estimates and their standard errors of both absolute and relative survival rates, and their relevance is shown for many of the commonest cancers.

In contrast to traditional age adjustment, the alternative method does not require performance of age-specific analyses, which often suffer from sparseness of data within age strata. Our analysis indicates that this conceptually and computationally simple method essentially provides the same point estimates with the same standard error as the traditional method for absolute survival rates (at least in situations where there is little loss to follow-up, as in the Finnish Cancer Registry), and more meaningful point estimates with an often smaller standard error for relative survival rates.

While the often substantially lower standard errors for relative survival constitute another major advantage of alternative age adjustment, a straightforward approach for their estimation has yet to be developed. In the meantime, however, derivation of such estimates by bootstrap as carried out in this analysis is an easily implementable alternative. Calculation of standard errors by bootstrap is to be recommended for relative survival rates anyway (even for crude analysis and for traditional age adjustment), given that standard errors of relative survival rates are often substantially overestimated by the conventional approach (Brenner and Hakulinen, 2005).

The empirical evaluation presented in this paper was restricted to ‘cohort analysis’ of survival, in which all patients included in the cohort could have potentially been followed over the full follow-up period of interest (here, 5 and 10 years), that is, in which there is no ‘technical censoring’. To explore the implications of technical censoring, we carried out additional ‘complete analyses’ of 5- and 10-year survival of patients diagnosed in 1989–1999 and followed until the end of 1999. With this type of analyses, traditional age adjustment, but not the alternative age adjustment, sometimes also substantially altered the values of absolute survival estimates, even when the age distribution of the standard population was the same as that of the study population. This is due to the fact that both the observed survival and censoring are age dependent and thus the censoring is informative. Usual care for informative censoring may have to be taken with the proposed method as with survival analysis methods in general.

In summary, application of the alternative method may enhance both validity and precision of comparative analyses of cancer survival.

## Figures and Tables

**Table 1:** Numbers and age distribution of patients diagnosed with common forms of cancer above 14 years of age in Finland in 1989 who were included in the analysis (the age distribution of patients in the EUROCARE-2 standard population is given for comparison)

	**Cancer patients in Finland 1989**			**EUROCARE-2 standard population**	
		**Proportion (%) in age group**		**Proportion (%) in age group**	
**Cancer site**	***N***	**15–44 years**	**75+ years**	**15–44 years**	**75+ years**
Oral cavity	416	8.9	32.7	8.8	18.1
Stomach	1039	4.3	41.3	3.3	39.2
Colon	970	6.3	41.8	3.6	39.6
Rectum	599	4.0	36.2	3.4	35.0
Pancreas	651	4.2	39.2	2.7	37.3
Lung	1909	2.0	25.5	2.1	25.5
Breast	2549	12.9	22.8	13.9	21.3
Cervix	159	20.1	32.1	36.6	11.1
Corpus	576	2.6	28.8	4.0	20.0
Ovaries	436	9.2	25.9	11.8	20.2
Prostate	1318	1.3^a^	48.9	1.6^a^	48.3
Kidneys	571	5.6	26.4	5.9	23.0
Urinary bladder	650	2.9	39.1	2.8	34.6
Melanoma	474	26.2	19.6	29.9	14.7
Thyroid gland	268	37.7	14.9	34.9	14.8

a Proportion in the age group 15–54 years.

**Table 2:** Five- and 10-year absolute and relative survival of patients diagnosed with common forms of cancer at ages 15–44 and 75+ years in Finland in 1989

	**Absolute**				**Relative**			
	**5 year-survival**		**10-year-survival**		**5 year-survival**		**10-year-survival**	
	**15–44**	**75+**	**15–44**	**75+**	**15–44**	**75+**	**15–44**	**75+**
Oral cavity	73.0	33.1	73.0	8.8	73.8	60.2	74.9	35.7
Stomach	23.0	9.6	23.0	5.6	23.3	17.3	23.7	22.3
Colon	77.0	27.7	72.1	15.1	77.7	47.7	73.6	54.6
Rectum	66.7	19.8	58.3	9.2	67.4	34.9	59.9	35.0
Pancreas	14.8	0.0	11.1	0.0	15.0	0.0	11.5	0.0
Lung	21.1	2.3	15.8	0.2	21.4	4.0	16.4	0.8
Breast	80.1	47.9	68.4	19.7	80.7	77.2	69.6	62.3
Cervix	81.3	15.7	81.3	7.8	81.8	25.6	82.5	26.0
Corpus	86.7	34.9	86.7	19.3	87.3	54.6	88.3	57.4
Ovaries	75.0	15.9	72.5	5.3	75.4	25.6	73.6	16.7
Prostate^a^	41.2	20.4	17.6	2.9	43.0	69.7	19.6	56.3
Kidneys	81.3	25.8	78.1	10.6	82.5	42.9	81.0	36.3
Urinary bladder	94.7	28.3	94.7	7.9	96.3	52.2	98.4	33.3
Melanoma	87.9	41.9	83.0	18.3	88.8	69.7	85.0	61.1
Thyroid gland	98.0	25.0	98.0	10.0	98.6	40.2	99.5	32.3

a Age classes are 15–54 and 85+ years.

**Table 3:** Crude and age-adjusted 5- and 10-year absolute survival of patients diagnosed with common forms of cancer above 14 years of age in Finland in 1989

	**5-year absolute survival**					**10-year absolute survival**				
		**Age adjusted**					**Age adjusted**			
		**PE**		**s.e.**			**PE**		**s.e.**	
**Cancer site**	**Crude**	**T**	**A**	**T**	**A**	**Crude**	**T**	**A**	**T**	**A**
Oral cavity	48.5	51.7	51.7	2.64	2.65	33.3	38.6	38.6	2.53	2.53
Stomach	17.1	17.3	17.3	1.16	1.17	13.0	13.2	13.1	1.03	1.03
Colon	40.2	39.7	39.7	1.55	1.54	29.9	29.4	29.4	1.42	1.41
Rectum	34.9	35.3	35.2	1.90	1.92	24.7	25.2	25.2	1.69	1.72
Pancreas	1.4	1.3	1.3	0.44	0.44	1.1	1.0	1.0	0.41	0.40
Lung	7.9	8.1	8.1	0.62	0.63	4.5	4.6	4.6	0.48	0.48
Breast	71.2	71.3	71.4	0.86	0.85	54.2	54.5	54.4	0.91	0.92
Cervix	49.0	62.6	62.6	3.99	3.99	42.1	57.2	57.3	3.95	3.92
Corpus	62.8	67.5	67.5	1.78	1.80	51.4	56.8	56.8	1.88	1.91
Ovaries	32.3	34.6	34.6	2.16	2.17	24.3	26.7	26.7	1.94	1.97
Prostate	42.6	42.8	42.8	1.36	1.35	19.1	19.1	19.1	1.06	1.06
Kidneys	44.8	46.3	46.3	2.02	2.02	32.6	34.3	34.3	1.88	1.88
Urinary bladder	49.2	50.6	50.6	1.82	1.85	31.6	33.2	33.2	1.66	1.67
Melanoma	71.3	73.5	73.4	1.88	1.90	59.0	62.1	62.0	2.03	2.04
Thyroid gland	77.2	74.9	75.0	2.26	2.23	72.8	70.4	70.4	2.25	2.21

Age adjustment is made to the site-specific EUROCARE-2 standard population. Point estimates (PE) and their standard errors (s.e.) obtained by bootstrap analyses with 10 000 replications are given for age adjustment by the traditional method (T) and by the alternative method (A).

**Table 4:** Crude and age-adjusted 5- and 10-year relative survival of patients diagnosed with common forms of cancer above 14 years of age in Finland in 1989

	**5-year relative survival**					**10-year relative survival**				
		**Age adjusted**					**Age adjusted**			
		**PE**		**s.e.**			**PE**		**s.e.**	
**Cancer site**	**Crude**	**T**	**A**	**T**	**A**	**Crude**	**T**	**A**	**T**	**A**
Oral cavity	61.5	61.2	61.1	3.04	3.13	54.5	52.9	54.8	3.59	3.58
Stomach	22.8	22.0	22.9	1.52	1.54	23.7	23.3	23.7	2.13	1.84
Colon	52.3	51.0	51.4	2.07	1.97	52.1	52.0	50.9	2.97	2.41
Rectum	45.0	43.9	45.1	2.49	2.46	42.4	40.9	42.6	3.29	2.89
Pancreas	1.8	1.4	1.7	0.47	0.56	1.9	1.2	1.8	0.46	0.69
Lung	10.0	9.5	10.2	0.75	0.79	7.5	6.3	7.6	0.68	0.79
Breast	80.8	80.6	80.7	1.05	0.95	70.8	69.6	70.6	1.47	1.18
Cervix	57.6	65.4	67.4	4.17	4.28	59.0	62.9	67.0	4.47	4.57
Corpus	73.7	75.0	76.5	2.06	2.03	73.0	72.6	74.8	2.71	2.49
Ovaries	37.3	37.9	39.1	2.42	2.45	33.0	31.6	34.7	2.52	2.56
Prostate	62.9	64.1	63.4	2.23	1.99	44.5	44.7	44.8	3.55	2.47
Kidneys	54.6	54.3	55.3	2.44	2.40	50.0	48.4	50.6	2.99	2.76
Urinary bladder	66.1	64.8	66.7	2.52	2.41	59.0	53.0	59.7	3.27	2.94
Melanoma	81.4	81.5	82.2	2.19	2.09	77.8	77.0	78.5	3.05	2.55
Thyroid gland	84.5	80.0	82.7	2.74	2.41	87.9	80.7	86.7	3.58	2.67

Age adjustment is made to the site-specific EUROCARE-2 standard population. Point estimates (PE) and their standard errors (s.e.) obtained by bootstrap analyses with 10 000 replications are given for age adjustment by the traditional method (T) and by the alternative method (A).
